# The molecular mechanism of ambrosin-induced cytotoxicity of human breast cancer and bladder cancer cells

**DOI:** 10.1016/j.jbc.2025.110531

**Published:** 2025-08-05

**Authors:** Layla El-Sawy, John R. Rubin, Luke Broses, Christopher Patsalis, Marian L. Henderson, Steven J. Wilson, Shelly A. Glase, Katherine A. Munson, Guadalupe Lorenzatti Hiles, Angelica Cates, Amir Emamdjomeh, Andrew Chou, Joseph Farber, Jeff W. Kampf, Brian Magnuson, Monica Liebert, Michelle T. Paulsen, Kenneth Van Golen, Mats Ljungman, Armand Bankhead, Phillip L. Palmbos, Kathleen C. Day, Mark L. Day

**Affiliations:** 1 Department of Urology, University of Michigan, Ann Arbor, Michigan, USA; 2 European Egyptian Pharmaceutical Industries, Alexandria, Egypt; 3 Rogel Cancer Center, University of Michigan, Ann Arbor, Michigan, USA; 4 Department of Computational Medicine and Bioinformatics, University of Michigan, Ann Arbor, Michigan, USA; 5 Department of Internal Medicine, University of Michigan, Ann Arbor, Michigan, USA; 6 Department of Chemistry, University of Michigan, Ann Arbor, Michigan, USA; 7 Department of Biostatistics, University of Michigan, Ann Arbor, Michigan, USA; 8 Department of Radiation Oncology, University of Michigan, Ann Arbor, Michigan, USA; 9 Department of Biological Sciences, University of Delaware, Newark, Delaware, USA

**Keywords:** bladder cancer, breast cancer, STLs, ambrosin, natural product, cancer therapy, cancer stem cells, glutamine, mitochondrial apoptosis

## Abstract

Sesquiterpene lactones (STL) are lipophilic compounds synthesized as secondary metabolites in species across the plant kingdom, most notably in the family Asteraceae. One STL, found in North African *Ambrosia maritima* (*A. maritima*), and Caribbean *Ambrosia hispida* (*A. hispida*), is the compound ambrosin. We have extracted ambrosin from *A. maritima* and *A. hispida* and demonstrated its toxicity in bladder cancer and breast cancer cell lines at low micromolar concentrations. Ambrosin also inhibited bladder cancer and breast cancer stem cell growth as non-adherent tumor spheroids. Sequencing of ambrosin-induced synthesis of nascent RNA revealed mitochondrial apoptotic gene signatures as well as activation of glutathione metabolism, indicating the generation of reactive oxygen species (ROS). Ambrosin exhibited antagonistic activity against EGFR tyrosine kinase and RhoC GTPase. Further studies showed that ambrosin inhibited EGFR auto-phosphorylation at tyrosine 1068 (Y1068) as well as the inhibition of RhoC GTPase activity. These findings indicate novel mechanisms of action and justify further considerations for the development of ambrosin as a potential agent for advanced bladder cancer and advanced breast cancer.

An estimated 82,000 new cases of bladder cancer are reported each year in the United States, with approximately one-quarter of these patients progressing to advanced muscle invasive and/or metastatic disease (1, 2). Patients with metastatic bladder cancer have been historically treated with chemotherapies, such as cisplatin and gemcitabine. More recently, patients with metastatic disease have been treated with immunotherapies that have exhibited some durable responses (3). However, current immunotherapies are only seeing success in a subset of eligible patients (4, 5). Thus, there remains a need to develop additional therapies for these patients.

Some breast cancers exhibit subtypes that share similar molecular landscapes with bladder cancer subtypes (6, 7). About 317,000 new cases of invasive breast cancer are diagnosed annually in women, with approximately 42,000 women dying from advanced breast cancer. A subset of advanced breast cancers are diagnosed as triple-negative breast cancer (TNBC), lacking expression of the estrogen receptor (ER), progesterone receptor (PR), and the Her2 tyrosine kinase receptor (1, 8). Treatments such as hormonal and trastuzumab-based therapy have shown success in receptor-positive breast cancer; however, many of these patients are not cured, and patients with TNBC are not eligible for these therapies (9). Thus, there is an urgent need for new treatments for advanced receptor-positive breast cancer and TNBC.

Various members of the plant kingdom synthesize chemical compounds (natural products) that have been used to treat a variety of diseases, including cancer (paclitaxel, vinblastine, *etc.*). These compounds often exhibit fewer adverse effects compared to their synthetic counterparts (https://chemistry-europe.onlinelibrary.wiley.com/doi/10.1002/cmdc.201800343). In our search for new treatments for metastatic bladder cancer and advanced breast cancer, we have examined cytotoxic compounds isolated from extracts of *Ambrosia maritima* and *Ambrosia hispida*. Among these compounds were sesquiterpene lactones (STL) that included ambrosin, damsin, damsinic acid, and neoambrosin.

Sesquiterpene lactones have gained appreciation for their medicinal utility. Dr Tu Youyou won the Nobel Prize in Medicine in 2015 for her research on artemisinin, an anti-malarial STL (10). Artemisinin targets protein alkylation, inhibiting multiple cellular pathways in *P. falciparum*, and, interestingly, in some tumor cells (11). Another STL, thapsigargin, is a potential chemotherapeutic that was in a clinical trial for the treatment of advanced solid tumors (NCT01056029). Thapsigargin may act more specifically by targeting the sarco/endoplasmic reticulum Ca2+ ATPase (SERCA) (12).

Over the past 5 decades, several studies have shown that ambrosin has cytotoxic effects on cancer cells at low micromolar concentrations (13, 14, 15, 16). In 1975, it was reported that ambrosin exhibited efficacy in mouse cancer xenograft models, but these data haves not been reproduced in mouse models since this publication (14). Little has been reported on the mechanism of action of ambrosin or its molecular targets in human cancer cells. However, a more recent study reported that ambrosin and a closely related family member, damsin, isolated from *Ambrosia arborescens,* target the NF-κB pathway, inhibiting the growth of breast cancer cells (16). In this study, we describe the isolation of ambrosin from two species of *Ambrosia*. Furthermore, ambrosin induces rapid transcriptional changes, inducing mitochondrial perturbation and apoptosis in bladder cancer and breast cancer cells and stem cells.

## Results

### Isolation and crystal structure of ambrosin from *A. maritima* and *A. hispida*

**Figure 1:**
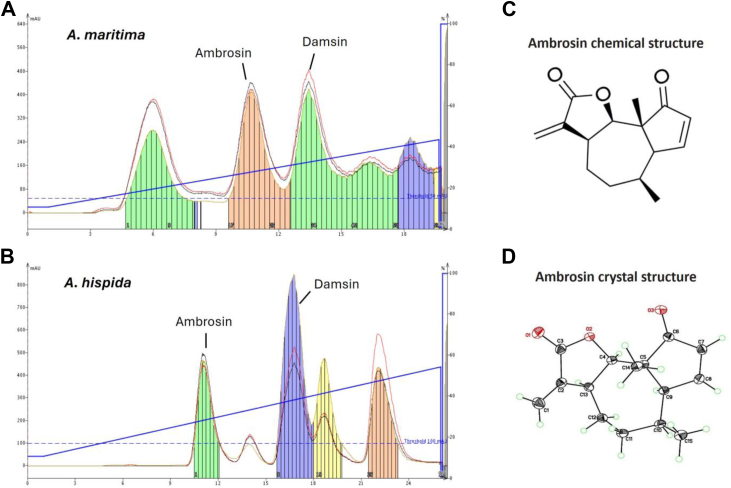
**Purification and identification of sesquiterpene lactones from *A. maritima* and *A. hispida***. *A* and *B* ethanol extracts prepared from *A. maritima,* and *A. hispida* were applied to HPLC-MS. Two major peaks were identified as ambrosin (MW 246.12) and damsin (MW 248.14). *C*, chemical Structure of Ambrosin. *D*, ORTEP Thermal Ellipsoid Plot of the Ambrosin Crystal Structure.

Two species of *Ambrosia* were the focus of an organic-based phytochemical extraction. The first was *A. maritima* (*A. maritima*), which is native to northeastern Africa (Egypt and Sudan). The second, *A. hispida* (*A. hispida*), is native to the Caribbean and, specifically, southern Florida in the United States. Sesquiterpene lactones (STLs) were extracted from 50 gm of dried leaves from *A. maritima* and *A. hispida* by reverse phase (C18) column chromatography, and ambrosin and damsin were identified by mass spectrometry (Fig. 1, *A* and *B*). The molecular weight of ambrosin is 246.30, and the calculated logP is 2.28. The chemical structure of ambrosin is shown in Figure 1*C*. The peak C18 chromatography fractions of ambrosin from extracts of *A. maritima* and *A. hispida* were subsequently re-chromatographed to 98% purity and allowed to crystallize. Blade-shaped crystals (2 × 2 × 4 mm) of ambrosin were obtained by slow evaporation from ethanol solutions. The crystals were determined to be orthorhombic, Space Group P212121, with unit cell dimensions of a = 7.911, b = 11.631, c = 13.969. Structures were solved by direct methods, and the absolute configuration was determined using full-matrix least squares refinement using anisotropic thermal parameters (R = 0.0305 for *A. maritima* and R = 0.0316 for *A. hispida*). The ORTEP plot crystal structure was also determined and provided (Fig. 1*D*). The crystal structures of ambrosin, whether isolated from *A. maritima* or *A. hispida*, were essentially identical based on the ORTEP thermal vibration plots. The structures were deposited in the Cambridge Crystallographic Data Centre (CCDC). (CCDC. https://www.ccdc.cam.ac.uk).

### Efficacy of ambrosin on metastatic bladder cancer and advanced breast cancer cell lines

**Figure 2:**
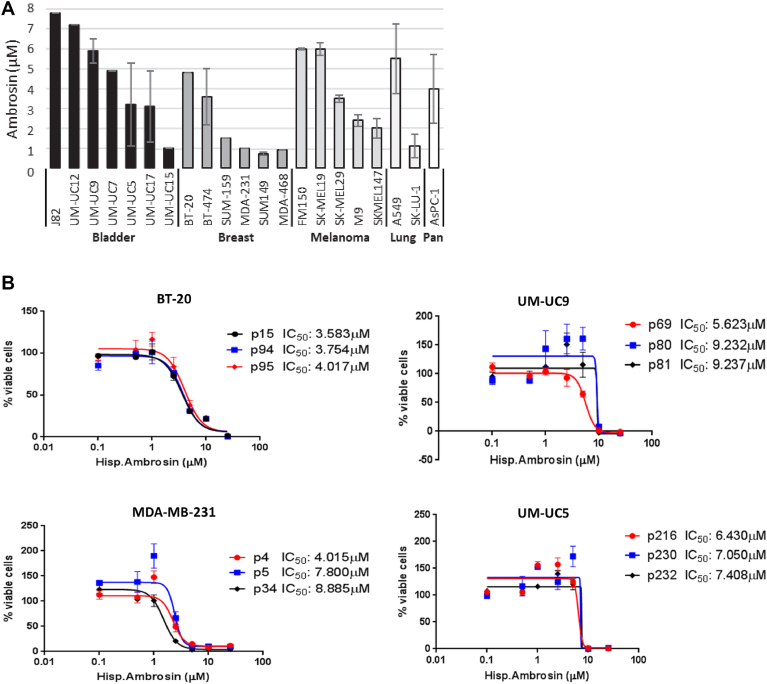
**Efficacy of ambrosin against multiple cancer cell lines**. *A*, MTS-generated cell viability values of ambrosin in bladder cancer cells, breast cancer cells, melanoma cells, lung cancer cells, and pancreatic cancer cells (Pan). The number of replicates per cell line ranged from paired to triplicate (standard deviations shown where appropriate). *B*, ambrosin IC_50_ values were determined for two triple negative breast cancer cell lines (BT-20 and MDA-231) and two advanced bladder cancer cell lines (UM-UC9 and UM-UC5) over three independent passages (p) of cells.

There is evidence that ambrosin has cytotoxic effects in several cancer models (13, 14, 15, 16). We performed MTS assays using ambrosin on 21 human cell lines representing bladder cancer, breast cancer, melanoma, lung cancer, and pancreatic cancer. The IC**_50_** values were calculated for all cell lines and were found to be in the range of 1 to 6 μM (Fig. 2*A*). Specifically focusing on bladder cancer and breast cancer, we calculated the IC**_50_** values in three different passages of two metastatic bladder cancer and two advanced breast cancer cell lines. IC**_50_** values exhibited close correlation between passages and cell lines, ranging from 4 to 9 μM (Fig. 2*B*). These findings demonstrate robust cytotoxicity of ambrosin in a variety of cancer cells, including metastatic bladder and advanced breast cancer cells.

### Efficacy of ambrosin on human bladder cancer and breast cancer stem cells

**Figure 3:**
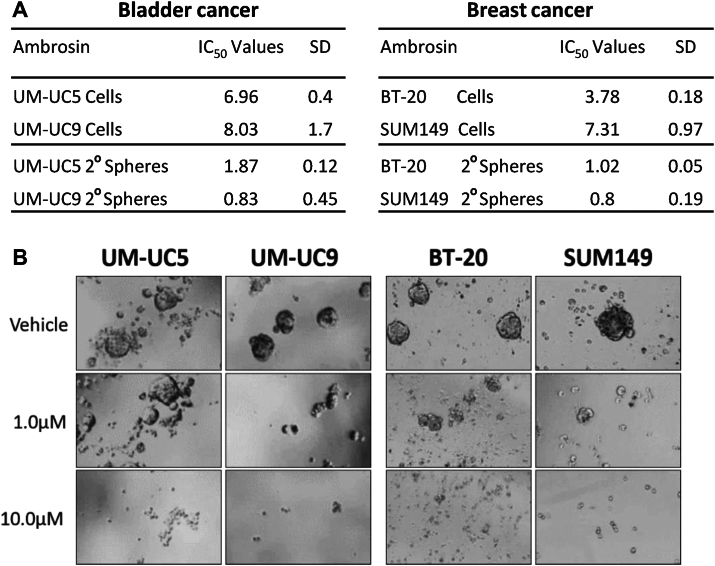
**Ambrosin targets the cancer stem cell population of advanced bladder cancer and triple negative breast cancer cell lines**. *A*, table of ambrosin IC_50_ values in μM for two bladder cancer cell lines and two breast cancer lines grown in normal adherent conditions and compared to IC_50_ values of these cell lines cultured in 3-D Matrigel spheres in Matrigel and determined by reduction in sphere-forming units. Standard deviation of the mean is presented (SD). *B*, representative images of ambrosin's effect on sphere viability in UM-UC5 and UM-UC9 bladder cancer spheres and BT-20 and SUM149 breast cancer spheres.

To investigate ambrosin toxicity in bladder cancer and breast cancer stem cells, we analyzed the sphere-forming efficiency (SFE) of secondary spheres of ambrosin-treated cells. To confirm that a CTC subpopulation is present in these cell lines, we evaluated expression of CSC genes in untreated and ambrsoin-treated bladder cancer and breast cancer cells. The CSC marker genes, ALDH1A, CD44, NANOG, SOX2, and SOX4 were all found to be expressed in these cell lines. Expression in RPKM (Reads Per Kilobase Million) revealed expression ranging from <1.0 RPKM for ALDH1A, SOX2 and NANOG to >2.0 RPKM for CD44 and SOX4. UM-UC5 and UM-UC9 bladder cancer secondary sphere formation was inhibited at IC_50_ concentrations of 1.87 μM and 0.83 μM, respectively. BT-20 and SUM149 breast cancer secondary sphere formation was inhibited at similar IC_50_ concentrations of 1.02 μM and 0.80 uM, respectively. Compared to adherent bladder cancer cells, SFE of secondary spheres were reduced 4-fold to 10-fold by ambrosin. SFE of BT-20 and SUM149 secondary spheres were reduced four-fold and eight fold respectivley compared to adherent cells. (Fig. 3*A*). Visual documentation was obtained by examining secondary spheres treated with vehicle or treated with 1 μM or 10 μM ambrosin for 48 h (Fig. 3*B*). These results suggest that ambrosin exhibits low micromolar toxicity against bladder cancer and breast cancer stem cells.

### Ambrosin induces rapid changes in cellular gene programs

**Figure 4:**
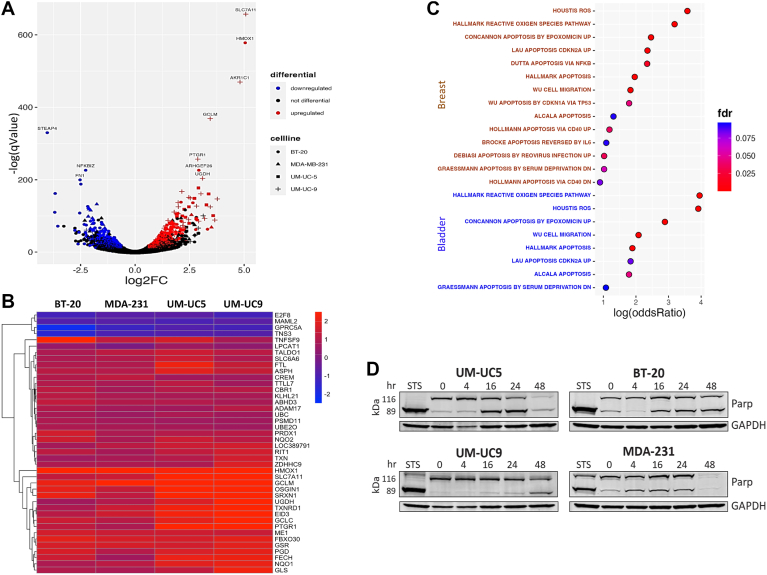
**Ambrosin induces mitochondrial apoptosis and PARP cleavage**. *A*, Volcano plot of differential expression results comparing samples treated with ambrosin for 4 h to their controls, respectively. Results for four cell lines are shown: BT-20, MDA-231, UM-UC5, and UM-UC9. Each gene is represented by four data points, one for the differential test performed in each of the four cell lines. A given gene is considered differentially expressed if it meets the criteria outlined in the methods. Upregulated DEGs are colored *red*, and downregulated DEGs are *blue*. *B*, A Heatmap showing log2(Fold Change) for all 42 common DEGs across the four cell lines. *C,* The dot plot represents the results of over-representation analysis (ORA) for pathways of interest in bladder and breast cancer cell lines. ORA was conducted for each cell line using the DEGs from differential expression analysis, respectively. *D*, Western blot of bladder cancer cells (UM-UC5, UM-UC9) and breast cancer cells (BT-20, MDA-231) treated with 10 μM ambrosin for the times indicated. Staurosporine (STS) treatment for 24 h was used as a positive control for PARP cleavage. GAPDH was used as a loading control.

To determine the molecular response to ambrosin, Bru-seq (17, 18) was employed to quantify nascent RNA expression after a 4 h treatment with ambrosin or its vehicle control. Two breast cancer and two bladder cancer cell lines were treated in parallel. Differential gene expression analysis was conducted to detect changes between vehicle and ambrosin treatment in each cell line. Significant overlap in differentially expressed genes (DEGs) following ambrosin treatment was observed in breast cancer cell lines (188 genes, pValue = 2.53e-139, odds ratio = 19.5) and bladder cancer cell lines (117 genes, pValue = 7.12e-99, odds ratio = 25.21) (Fig. 4*A*). Total DGE and overlap of DE genes in breast cancer lines and bladder cancer lines is shown in Figure S1*A*. The top 100 genes that were differentially expressed in all four cell lines are also provided (Fig. S1*B*). All four cell lines exhibited a set of 42 common DEGs in response to ambrosin treatment (Fig. 4*B*). Over-representation analysis was also performed to elucidate the biochemical processes enriched in response to ambrosin exposure. The set of DEGs for each cell line was used to test pathways in the KEGG, GO, and MSigDB pathway databases for enrichment. In all, 1127 and 717 pathways were found to be significantly enriched in breast cancer and bladder cancer, respectively (FDR < 0.05). One hundred eighty-four of the aforementioned pathways were enriched in both breast and bladder cancer. In both bladder and breast cancer cell lines, ambrosin upregulated gene sets for mitochondrial apoptosis as well as glutathione metabolism, and downregulated genes involved in extracellular matrix organization and regulation of cellular migration (Fig. 4*C*). These findings indicate that ambrosin induces the differential expression of anti-tumor gene signatures characterized by changes in several important cellular functions.

### Mechanism of ambrosin cytoxicity

Mitochondrial apoptosis is a likely mechanism of ambrosin cytotoxicity, as suggested by the pathway analysis of the ambrosin-associated transcriptional signatures (Fig. 4*C*). Several genes within these sets associated with mitochondrial apoptosis function to regulate mitochondrial membrane integrity, mitochondrial ROS production, and mitochondrial programmed cell death (19, 20, 21, 22, 23, 24).

A key marker of mitochondrial apoptosis is the activation of caspases and the proteolytic cleavage of poly(ADP-ribose) polymerase (PARP) from the 116 kDa precursor to a 89 kDa cleavage product (25). UM-UC5 and UM-UC9 bladder cancer cells and BT-20 and MDA-231 breast cancer cells were treated with 10 uM ambrosin over a 48 h time course before cellular proteins were isolated for Western blotting and probed with anti-PARP and anti-GAPDH antibodies. Ambrosin-treated cells exhibited a reduction in the 116 kDa precursor protein with a concominant increase in the 89 kDa fragment of PARP over 48 h. Maximum cleavage was observed by 24 h (Fig. 4*D*). Given the correlation between the transcriptional signature of mitochondrial apoptosis and the functional cleavage of PARP induced by ambrosin, mitochondrial apoptosis is a likely mechanism of ambrosin cytotoxicity in breast and bladder cancer cells.

### Ambrosin inhibits EGFR phosphorylation

**Figure 5:**
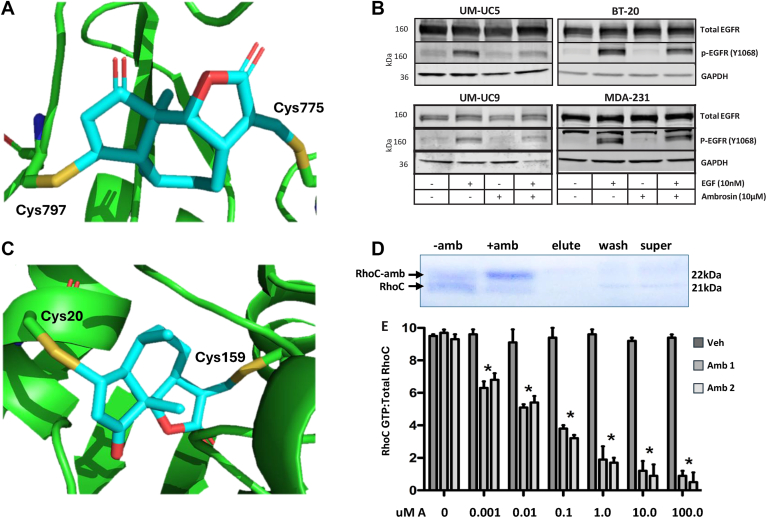
**Inhibition of EGFR and RhoC following ambrosin treatment**. *A*, detail of the molecular model of ambrosin (*cyan*) bound to Cys 775 and Cys 797 in the ATP binding site of EGFR kinase domain. *B*, the indicated cells were treated with vehicle, 10 nM EGF, 10 uM ambrosin or 10 nM EGF plus 10 uM ambrosin for 48 h. Antibodies for total (unphosphorylated) EGFR and Y1068 phosphorylated EGFR (p-EGFR Y1068) were used to probe the Western blot. GAPDH antibody was used as a protein loading control. *C*, details of the molecular model of ambrosin (*cyan*) binding Cys 20 and Cys 159 in the guanosine nucleoside binding site of RhoC. *D*, Ambrosin bound RhoC (**+**amb) isolated by affinity chromatography exhibited an increased molecular weight shift compared to unbound RhoC (-amb) by acrylamide/SDS gel electrophoresis. Blank column elutes, wash, and pre-column supernatant are shown. *E*, active RhoC was detected using a GTP-bound specific antibody form breast cancer cells treated with increasing concentrations of ambrosin for 24 h. Ethanol was used as vehicle control. The ratio of active to total RhoC GTPase is presented. The ethanol vehicle control (*dark bars*) is compared to treatment of cells with two separate preparations of ambrosin (*lighter bars* amb 1 and amb 2) and presented as mean ± standard error. Quantitation is presented as arbitrary units (A.U.) and significant inhibition of RhoC activity denoted (∗) *p* < 0.05.

One of the genes downregulated in the MSIgDB cellular migration set was epidermal growth factor receptor (EGFR). EGFR was differentially expressed in response to ambrosin in bladder cancer and breast cancer cells. Even though EGFR expression was downregulated in both breast cancer lines and one bladder cancer cell line (data not shown), EGFR activity is traditionally determined by its state of phosphorylation at specific tyrosine residues. Molecular modeling methods indicated covalent binding to cysteine 775 and cysteine 797 in the ATP binding pocket of the EGFR kinase domain that would inhibit ATP binding and auto-phosphorylation activity (Fig. 5*A*). To determine if ambrosin inhibited EGFR phosphorylation, we stimulated serum-deprived bladder cancer and breast cancer cells with 10 nM EGF for 2 h to induce auto-phosphorylation of tyrosine 1068 (Y1068), a documented marker of EGFR activation. This was followed by treatment with 10 μM ambrosin for 6 h. Total cell protein was subjected to Western blotting probed with a phospho-Y1068 specific antibody, and revealed substantial inhibition of EGFR phosphorylation at Y1068 in the bladder cancer cell lines and slightly less inhibition in the breast cancer cell lines (Fig. 5*B*). These findings indicate ambrosin likely binds to the tyrosine kinase domain of EGFR, directly inhibiting phosphorylation at Y1068 in bladder cancer cells and breast cancer cells.

### Ambrosin inhibition of RhoC activity

Cytoskeletal regulation and cell migration were two GO categories implicated in ambrosin treatment. To corroborate these signatures, we demonstrated that ambrosin significantly inhibited tumor cell transmigration by 75% using a matrigel transwell assay (Fig. S2). Rho GTPases are critical mediators of both processes and have been implicated in the invasive progression of breast cancer and bladder cancer (26, 27, 28). RhoC is regulated by several tryosine kinases, including EGFR, that mediate cytoskeletal and migratory functions (29). RhoC cycles between the active GTP-bound and inactive GDP-bound forms of the enzyme (30). Molecular modeling indicated covalent binding of ambrosin to cysteine 20 and cysteine 159 in the guanine nucleotide binding pocket of RhoC. This binding mode would inactivate RhoC by blocking the binding of GDP or GTP. (Fig. 5*C*). Experimental confirmation of ambrosin binding to RhoC was obtained by the addition of ambrosin to a RhoC affinity column and eluting the RhoC complexes. Analysis of the eluted complex by acrylamide gel electrophoresis showed an upward shift in molecular weight of RhoC, indicative of RhoC/ambrosin complex formation (Fig. 5*D*).

To examine ambrosin's effect on RhoC activation, we employed a modified G-LISA assay to selectively isolate the active GTP-bound form of RhoC from ambrosin-treated and control-treated cells. Using this assay, we determined that two separate preparations of ambrosin effectively inhibited GTP binding to RhoC (Fig. 5*E*). This result indicates that ambrosin may sterically prevent the binding of GTP, stabilizing RhoC in its inactive form.

### Detoxification of ambrosin by glutathione

**Figure 6:**
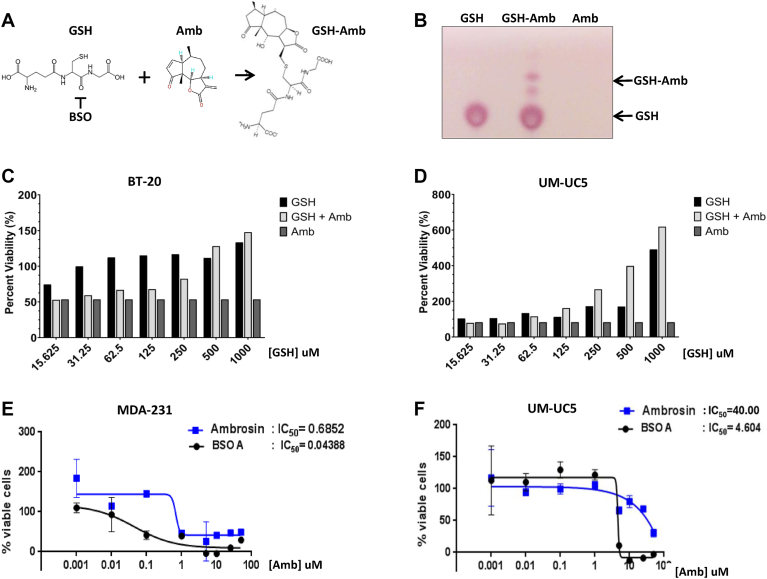
**Ambrosin is inactivated by glutathione**. *A*, chemical pathway for conjugation of free glutathione (GSH) and ambrosin (Amb) resulting in the inactive GSH-Amb conjugate. Buthionine sulfoximine (BSO) inhibits the production of GSH. *B*, Thin-layer chromatography of free GSH and the GSH-Amb conjugate chromatographed for 24 h. *C, D*, aAmbrosin toxicity is neutralized by GSH as measured by MTS assays of BT-20 and UC5 cells treated with 20 μM ambrosin for 48 h, in combination with an increasing concentration of GSH and normalized to 50% viability for ambrosin alone. *E, F*, inhibition of GST synthesis by pretreatment with 100 uM BSO increased ambrosin cytotoxicity (IC50) in UM-UC5 bladder cancer cells and MDA-231 breast cancer cells over a 100,000-fold dose escalation of ambrosin and measured by MTS assay.

Gene set pathway analysis revealed enrichment of cellular ROS production and glutathione metabolism, indicative of increased glutathione (GSH) synthesis in response to ambrosin treatment. The chemical reaction of GSH and ambrosin predicts thiol binding of GSH to the free oxygen electrophile of ambrosin (Fig. 6*A*). This interaction was supported by the demonstration of GSH and ambrosin complex formation resolved by silica gel chromatography (Fig. 6*B*). Furthermore, the addition of exogenous glutathione reduced ambrosin cytotoxicity in both breast cancer and bladder cancer cells, as measured by MTS viability assays (Fig. 6, *C* and *D*). Additionally, co-administration of ambrosin with buthionine sulfoximine (BSO), a known inhibitor of GSH synthesis (23, 31), sensitized breast cancer and bladder cancer cells to ambrosin cytotoxicity by 15- and 10-fold, respectively (Fig. 6, *E* and *F*). These findings indicate that ambrosin-treated cells induce GSH synthesis as a detoxification response to increased ROS production and, consequently, represent the mechanism of ambrosin resistance in cancer cells.

## Discussion

Two species of *Ambrosia*, North African *A. maritima* and Caribbean *A. hispida,* were the source of organic-based phytochemical extractions of sesquiterpene lactones to test basal antitumor activity in human cancer cell lines. The primary extracts from these sources were cytotoxic in multiple human cancer cell lines at low μg/ml concentrations.

Based on these results, we developed a reverse-phase chromatography and mass spectrometry (MS) protocol to isolate and identify several sesquiterpene lactones (STLs) from organic extracts of *A. maritima* and *A. hispida*. Based on the MS signatures, one of these compounds was believed to be the STL ambrosin. Confirmation of ambrosin was achieved by X-ray diffraction and ORTEP crystal structure determination, which were essentially identical whether isolated from *A. maritima* or *A. hispida*.

Ambrosin was selected for further investigation in multiple cancer cell lines representing five different tumor types. More specifically, ambrosin's effect on two advanced bladder cancer cell lines and two triple-negative breast cancer cell lines was more thoroughly characterized. Ambrosin exhibited cytotoxicity in all cell lines at IC**_50_** values of 1 to 8 μM. These data suggest that tumor cell toxicity observed in *A. maritima* and *A. hispida* extracts is due, in large part, to ambrosin purified from these plants.

Cancer stem cells are thought to exist in all tumors and may be responsible for resistance to radiation, chemotherapy, and possibly targeted therapy (32). Thus, cancer stem cells are a promising target for emerging anti-cancer therapeutics. To investigate cancer stem cell cytotoxicity of ambrosin, we analyzed primary and secondary sphere formation of bladder cancer and breast cancer stem cells. Ambrosin exhibited 3.5- to 10-fold greater efficacy against secondary sphere formation in both bladder cancer and breast cancer cells compared to primary cell lines. These results suggest that ambrosin is cytotoxic to primary adherent bladder and breast cancer cells but is significantly more toxic to the CSC population.

Employing RNA Bru-seq, we examined the nascent expression of RNA following a four-hour ambrosin treatment window in bladder cancer and breast cancer cells. Analysis of these data provided important information regarding ambrosin's mechanism of action and mechanism of ambrosin resistance. Differential expression and pathway analysis reveal that ambrosin treatment in the four cell lines exhibited a transcriptional signature that is characterized by upregulation of anti-tumor cellular processes, such as glutathione production and apoptosis. The analyses suggest restricted yet mutual cellular responses to ambrosin, as they are consistent in all cell lines.

Mitochondrial apoptosis is a likely mechanism of ambrosin toxicity, as suggested by the pathway analysis of DEG signatures in each cell line, respectively. Nine of the 161 genes in the apoptosis gene set were independently significantly differential, including the downregulation of the anti-apoptotic gene, BCL2L1. BCL2L1 protein is located at the outer mitochondrial membrane and has been shown to regulate mitochondrial membrane potential and the production of ROS, resulting in oxidative apoptosis (33, 34).

Two mitochondrial pro-apoptotic genes were also induced by ambrosin in breast cancer and bladder cancer cells. Mitochondrial apoptosis-inducing factor (AIFM2) is reported to have pro-apoptotic function (19) and is also associated with the outer mitochondrial membrane, where it functions as an oxidoreductase in apoptotic signaling. AIMF2 was downregulated in many cancer cell lines compared to non-tumor cell lines from the same tissues (35) but can induce apoptosis when overexpressed in cancer cells (36). The second over-expressed pro-apoptotic gene was peptidyl-prolyl cis-trans isomerase F (PPIF), which regulates mitochondrial membrane permeability and cell death (20, 21, 22). PPIF is a component of the mitochondrial permeability transition pore (mPTP) and may function in sustained mPTP opening, leading to disruption of the outer mitochondrial membrane, increasing ROS levels, caspase activation, and apoptosis (37, 38, 39).

The reduction of BLCL2L1 expression and increased expression of AIFM2 and PPIF in response to ambrosin indicate apoptosis by mitochondrial disruption or signaling. The release of AIFM2 is associated with caspase-dependent apoptosis and the proteolytic cleavage of poly(ADP-ribose) polymerase (PARP) (25). We demonstrated that ambrosin-treated breast cancer cells and bladder cancer cells exhibited PARP cleavage from its 116 kDa precursor to the 89 kDa product starting at 4 to 16 h, temporally aligning with ambrosin's rapid induction of the mitochondrial apoptotic signature.

Another cellular process induced by ambrosin treatment was the rapid induction of genes that regulate glutathione synthesis. These genes encode dimeric subunits of the glutamate cysteine ligase (GCL), which consists of the catalytic subunit (GCLC) and a modifier subunit (GCLM). These genes play key roles in the synthesis of glutathione (GSH) in response to mitochondrial ROS production and are the main mechanism of chemoresistance in cancer patients (23). Activation of the glutathione pathway by ambrosin suggests a mechanism of cellular resistance that is confirmed by the finding that glutathione binds to ambrosin, as demonstrated by chromatography. Furthermore, the addition of recombinant GSH was sufficient to reduce ambrosin cytotoxicity in cultured cells. However, the addition of buthionine sulfoximine (BSO), a known inhibitor of GLC and GSH synthesis (23, 31), dramatically increased ambrosin cytotoxicity. These results are unsurprising, as glutathione is known to detoxify electrophilic Michael acceptors, of which ambrosin has two (40).

Cell migration was another regulatory pathway down regulated by ambrosin. While this pathway represents a large cohort of regulatory genes, we selected the EGFR gene for further study due to its role in bladder cancer and breast cancer cell migration and invasion during metastatic progression (41, 42, 43, 44, 45). Molecular modelling indicated that ambrosin can bind in the ATP binding pocket of the EGFR kinase domain forming covalent bonds to two adjacent cysteines. This binding mode would inhibit ATP binding and, thus, EGFR kinase activity. This hypothesis is consistent with ambrosin's inhibition of EGF-induced auto-phosphorylation of the critical Y1068 residue of EGFR in bladder cancer cells and, to a lesser extent, in triple negative breast cancer cells. In agreement with our results, Fan *et al.* had previously reported that ambrosin induces apoptosis of MDA-231 cells with associated repression of BcL2 and inhibition of AKT and GSK-3β phosphorylation (46). While we did not specifically examine the AKT/GSK axis, it is known that EGFR is an upstream regulator of AKT and that inactivation of EGFR by ambrosin would likely inhibit the phosphorylation of AKT and GSK-3β (46). Another consequence of EGFR inhibition by ambrosin is suggested by Sotillo *et al.* who demonstrated that ambrosin inhibited nuclear translocation of p65/NF-κB, which is reciprocally regulated by EGFR (16, 47). While the Sotillo study did not examine EGFR, we can partially corroborate their findings through our GSEA analysis, which identified transcriptional changes in several NF-κB pathway gene sets. We also corroborated their finding that p65 protein expression is inhibited by ambrsoin in a different breast cancer cell line, BT-474 (Fig. S3).

Due to EGFR's role in the regulation of RhoC during tumor cell migration and invasion, RhoC GTPase was selected for further study. Molecular modeling indicated that ambrosin can bind adjacent to the guanosine nucleotide binding site of RhoC, thereby blocking guanine nucleotide access to the RhoC active site. This was confirmed experimentally by the finding that ambrosin effectively inhibits the binding and formation of the activated (GTP) form of RhoC.

To conclude, we have determined that ambrosin exhibits cytotoxicity against five different types of cancer cells at low micromolar concentrations. Specifically focusing on advanced bladder and breast cancer, we have determined some of ambrosin's mechanisms of action. This includes mitochondrial apoptosis, ROS production, and the mechanism of ambrosin detoxification. We have also identified specific enzyme targets of ambrosin, such as EGFR and RhoC, which are of interest due to their role in the metastatic progression of several tumor types. This study has provided an in-depth molecular platform from which to continue preclinical analysis of ambrosin for therapeutic development in the treatment of metastatic bladder cancer and advanced breast cancer. Another potential target of ambrosin had been identified by Sotillo *et al.,* who had reported that ambrosin inhibits the translocation of p65/NF-κB and increases the expression of p65 protein in breast cancer cells. We found that NF-κB gene sets were induced by ambrosin in our GSEA analysis of bladder cancer and breast cancer cells, and that the expression of p65 protein is also induced by ambrosin in breast cancer cells, indicating that the NF-κB axis may also be a therapeutic target for further ambrosin studies. It is noteworthy that ambrosin does exhibit several desirable pharmacologic characteristics, including compliance with the Rule of Five (RO5). However, Drug Metabolism and Pharmacokinetics (DMPK) studies should be considered in future studies of ambrosin drug candidacy.

## Experimental procedures

### Plant material

Samples of *A. maritima* were procured from various sites in Egypt and Sudan and were verified by L. El-sawy at EEPI in Alexandria, Egypt. *A. hispida* samples were collected from a site in Monroe County, Florida (GPS coordinates 24° 32′ 45″ N, 81° 48′ 30″ W). The sample was identified by Dr Alan Franck (Faculty of CMMB, University of South Florida), and the voucher specimen (286,382) deposited in the University of South Florida Herbarium.

### Extraction and purification of STLs from *A. maritima* and *A. hispida*

*A. maritima* and *A. hispida* leaves were dried and pulverized. 50 gm of the resulting powder was extracted with 500 ml of ethanol at room temperature for 4 days. The liquid extract was filtered through a 0.22 μm corning filter and evaporated to dryness and the recovered powder (1.55gm) was redissolved in 20 ml of ethanol to a final concentration of 77.5 mg/ml. 10 ml of ethanol extract (775 mg) was loaded onto a 60 g Biotage C-18 reverse phase column on a Biotage Isolera chromatography system (Biotage Inc.) and compounds eluted with a gradient (10–43%) of acetone in water. Eluted compounds were monitored by UV absorption at 211 and 219 nm. Ambrosin and Damsin were eluted as clearly distinct peaks (Fig. 1*A*) and identified by mass spectrometry. Peak fractions were evaporated to dryness and resuspended in ethanol, yielding 90.8 mg (11.7%) for ambrosin and 124.5 mg (16.1%) for damsin from a starting mass of 775 mg. Ambrosin was further purified by recrystallization from ethanol.

### Crystallization and crystal structure determination

Colorless crystals of ambrosin were obtained by slow evaporation of ethanol solutions of C18 column peak fractions. X-ray diffraction data were collected at reduced temperature (85 K) on a Rigaku AFC 10K Saturn 944+ CCD-based diffractometer equipped with a Cu targeted micro focus rotating anode X-ray generator. The structures were solved by direct methods (SHELXTL) and refined by full-matrix least squares to a residual R-factor of 0.0305. Ambrosin extracted from *A. maritima* and *A. hispida* produced identical atomic structures (Fig. 1*D*).

### Cell culture

A549, AsPC-1, BT-20, BT-474, J82, MDA-231, MDA-468, SK-LU-1, SUM149, and SUM159 cell lines were purchased from American Type Culture Collection (ATCC). All UM-UC- cell lines (UC-5, 7, 9, 12, 15, or 17) were obtained from their originator, Dr Grossman, at MD Anderson Cancer Center, Texas. These cell lines were fingerprinted using Cell Check 9 (IDEXX) and regularly checked for *mycoplasma* infection with PlasmoTest (InvivoGen). For all experiments with triple-negative breast cancer cells, the culture medium was Ham's F12 supplemented with 50 μM hydrocortisone, ITES (Biowhittaker), and 5% fetal bovine serum (FBS). Bladder cancer cell lines were propagated in Dulbecco's Modified Eagle Medium (DMEM) supplemented with 10% FBS and 15 to 1000 μM glutathione.

### Cell line and stem efficacy analysis

For each cell line, 1000 cells were seeded per well into 96-well plates where each well contained DMEM with 10% FBS. After cells were incubated for 24 h, drugs were added to reach the indicated concentrations and incubated for a further 96 h 40 μl of MTS/PMS solution were added to each well, and the absorbance at 490 nm was measured after 2 to 4 h of incubation at 37 °C (48). Blank wells, wells with medium alone and wells with medium plus 2.5 μM Staurosporine (Sigma-Aldrich Co.) were included as controls. Cell viability (%) was assessed as: (OD sample–OD Staurosporine)/(OD medium–OD Staurosporine) × 100.

### Bromouridine labeling, isolation of bromouridine-labeled RNA, and cDNA library preparation

Bru-seq was performed as previously described (18, 49) in triplicate on the following treated cell lines. Bladder cancer (UM-UC-5, UM-UC-9) and breast cancer (BT-20, MDA-231) cell lines were cultured with either vehicle (ethanol) or 10 μM ambrosin for 3 h. Bromouridine (Bru) (Aldrich) was added to the culture media to a final concentration of 2 mM, and cells were incubated at 37 °C for 30 min. Cells were lysed in Trizol, total RNA was isolated, denatured, and added to anti-bromodeoxyuridine conjugated magnetic beads. This process allowed for the capture and elution of the Bru-RNA. The cDNA libraries were prepared using Illumina's TruSEQ RNA Library Prep Kit, with the exclusion of polyA RNA isolation and the first and second-strand cDNA synthesis. Sequencing the cDNA libraries prepared from nascent RNA was performed at the University of Michigan Sequencing Core using the Illumina HiSeq 2500 sequencer according to manufacturer guidelines.

### Bioinformatics analysis

Sequenced reads were processed, mapped, and quantified per gene using the Bru-seq data analysis pipeline (18). Differential expression was calculated using DESeq2 v1.18 (50) and significant changes were required to have |fold change| >1.5, FDR-adjusted *p*-value < 0.01, RPKM > 0.5, and gene length > 300 bases. Differentially expressed genes were tested for gene set enrichment using gene sets sourced from the GO.db R package v3.3.0 and KEGG (51, 52) using a Fisher's exact test FDR-adjusted *p*-value threshold < 0.1 for significance. Each cell line was analysed independently in differential expression and subsequent pathway analyses. Graphing was performed in the R statistical programming language using the ggplot2 and pheatmap R packages.

### RhoC GTPase activation assay

Cells were grown to 80 to 90% confluence and harvested prior to sample preparation. Sample preparation and assay procedure were followed as per the manufacturer's protocol. RhoC activation assay kit (ab211166) (Abcam, Cambridge) was used, which provides Rhotekin RBD Agarose beads to selectively isolate and pull down the active form of Rho from purified samples. Endogenous active and total RhoC GTPase was determined using a G-LISA RhoA activation assay kit and a Total RhoA ELISA kit (Cytoskeleton, Inc.) with previously described modifications (53) (https://onlinelibrary.wiley.com/doi/10.1002/ijc.25655). Briefly, SUM149 cells were grown to 75% confluence under normal growth conditions in 100 mm dishes (26). A dose range of ambrosin, 1 nM-100 μM, was added directly to the cultured cells and incubated for 24 h. Ethanol was used as a vehicle control. Protein lysates were harvested and G-LISA/ELISA performed per manufacturers' instructions, with the exception that a chicken anti-RhoC, developed by our laboratory, was used and detected with a goat anti-chicken HRP secondary antibody (Abcam, Cambridge, MA).

### Sphere viability

Primary and secondary spheres were developed as previously described (54). Ambrosin (10 μM), was added to the cultures at the time the secondary spheres were plated. Analysis was completed at the end of 5 days of growth in MammoCult media.

### Glutathione/ambrosin conjugation and thin-layer chromatography

Ambrosin (12.5 mM) and glutathione (50 mM) were incubated in phosphate buffer saline (PBS), pH 7.4, for 1 h. Thin-layer chromatography of the reaction products indicated the formation of glutathione-ambrosin conjugates.

### Invasion assay

Cells were harvested and seeded at 6.25 × 10^5^ cells per well in a Corning Matrigel-coated 24-well plate invasion chamber at 0.8 microns in serum-free media. Matrigel inserts were rehydrated with serum-free media and incubated until ready to use. 500 μL of serum-containing media was added to the bottom chamber, and 200 μL of ambrosin-treated cells (0, 1, or 100 μM of ambrosin) (four wells per concentration) was added into the matrigel-coated insert and incubated for 24 h. After incubation, the media was aspirated from both the filter and the bottom well chamber. The Matrigel was removed from the filter using a cotton swab. Next, the filter and bottom well chamber were stained with crystal violet for 30 min, washed with water, and dried overnight. Stained cells were visualized using bright-field microscopy.

### Western blotting and antibodies

Western blotting was carried out as previously described (55). For phosphorylated proteins, the blocking buffer was TBST with 5% bovine serum albumin. Blots were probed with the following primary antibodies and their specificity described in the commercial technical data: anti-EGFR (ThermoFisher MS-665-P), anti-PARP (Cell Signaling Technologies #9542), anti-Phospho-EGFR Y1068 (Cell Signaling Technologies #2234), and anti-GAPDH (Abcam ab181603). Blots were visualized using LICOR, and images were analyzed using Image Studio v5.2.

### Statistical analysis

Results of *in vitro* experiments are presented as mean ± standard deviation or mean ± standard error. The paired student *t* test was used to compare continuous variables when there were two groups.

## Data availability

Crystal parameters and atomic coordinates for the ambrosin crystal structure were deposited in the Cambridge Structural Database (deposition number 1882557). Gene expression data are available at the Gene Expression Omnibus (accession GSE293803).

## Supporting information

This article contains supporting information. Figure S1, Figure S2, Figure S3.

## Conflict of interest

The authors declare the following financial interests/personal relationships which may be considered as potential competing interests: M. Day was a paid consultant for EEPI on this study. All other authors declare no conflict of interest.
