# Supplementary material for: The molecular mechanism of ambrosin-induced cytotoxicity of human breast cancer and bladder cancer cells
Source: J Biol Chem. 2025 Aug 5;301(9):110531. doi: 10.1016/j.jbc.2025.110531 (PMC12409444; doi:10.1016/j.jbc.2025.110531)
Supplement: Supplemental Figure legends [file mmc1.docx]

**Supplemental figure legends.**

**Supplemental figure 1. Overlapping and top 100 DE genes induced by ambrosin.** (A) Overlaping DE genes between 2 breast cancer lines and and 2 bladder cancer lines. (B) heat maps of the top 100 DE genes in all four cell lines.

**Supplemental figure 2.** **Ambrosin inhibits the invasion of breast cancer cells.** (A) SUM 149 breast cancer cells were untreated, treated with ethanol vehicle (Veh Ctl), or treated with 1uM or 10uM ambrosin (Amb) and placed into matrigel coated transwell chambers for 24 hours. Crystal violet-stained cells were visualized on the underside of the membrane using bright field microscopy to count invasive cells. Bars represent mean ± SEM (N = 3) p<0.05 *.

**Supplemental figure 3. Western blot of p65 expression following treatment with ambrosin and damsin.** Breast cancer cells (BT-474) were treated with vehicle control (control), 10ug organic *A. maritima* extract (organic), 10 µM ambrosin or 10 µM damsin for 4 hours and resulting lysates run on western blots probed with anti-human p65 antibody. GAPDH was used as a loading control from same lysate. Bottom graph represents Lycor densitometry of western blot in top panel.
