# Supplementary figures and images for: The molecular mechanism of ambrosin-induced cytotoxicity of human breast cancer and bladder cancer cells
Source: J Biol Chem. 2025 Aug 5;301(9):110531. doi: 10.1016/j.jbc.2025.110531 (PMC12409444; doi:10.1016/j.jbc.2025.110531)

**A**

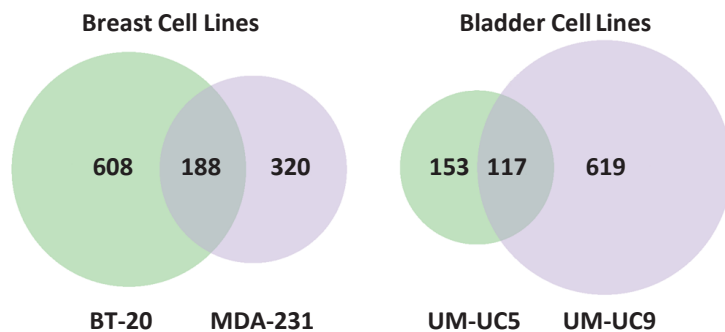

**B**

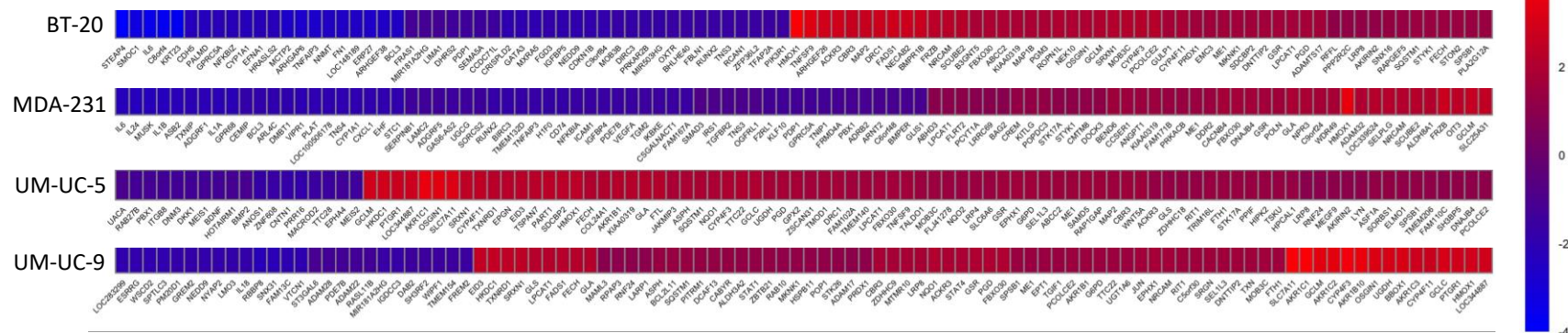

Supplement: Figure S1 [file mmc2.pdf]

Supplemental  
figure 2

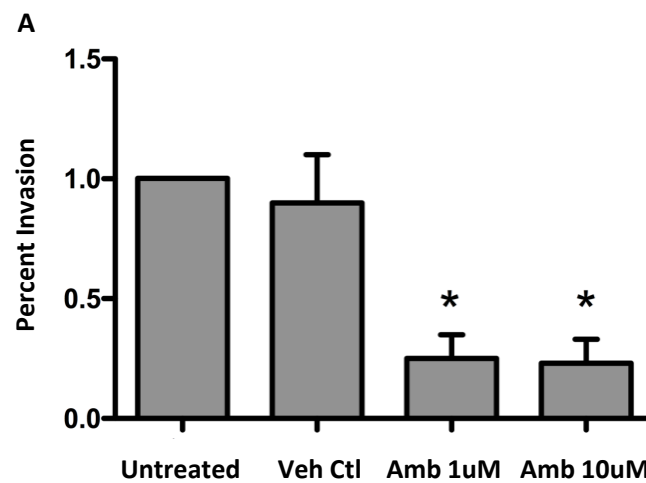

Supplement: Figure S2 [file mmc3.pdf]

**Supplemental  
figure 3**

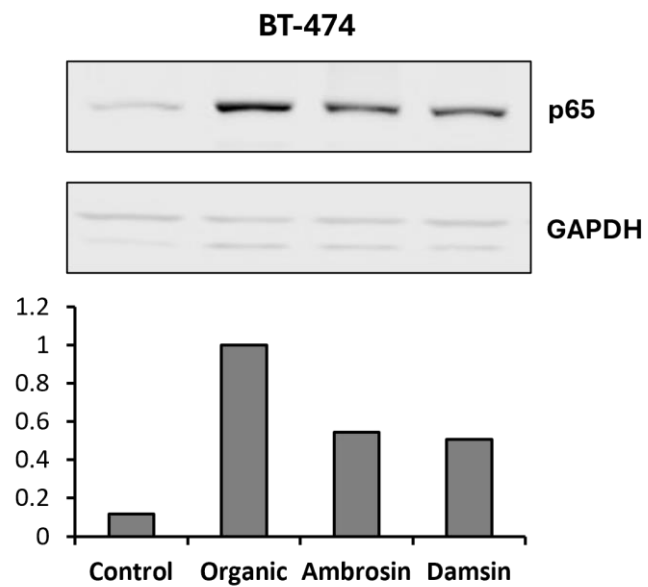

Supplement: Figure S3 [file mmc4.pdf]
